# First Testing of Literature-Based Models for Predicting Increase in Body Weight and Adipose Tissue Mass After Kidney Transplantation

**DOI:** 10.1177/15269248221122961

**Published:** 2022-09-02

**Authors:** Gabriela Schmid-Mohler, Sonja Beckmann, Patrizia Zala, Laura Huber, Ulrike Held, Thomas Fehr, Rudolf Peter Wüthrich, Heidi Petry, Thomas F. Mueller

**Affiliations:** 1 Division of Nephrology, 27243 University Hospital Zurich, Zurich, Switzerland; 2 Center of Clinical Nursing Science, 27243 University Hospital Zurich, Zurich, Switzerland; 3 Department of Biostatistics at Epidemiology, Biostatistics and Prevention Institute, 27217 University of Zurich, Zurich, Switzerland; 4 Department of Internal Medicine, Cantonal Hospital Graubünden, Chur, Switzerland

**Keywords:** clinical outcomes, behavior, addictive, clinical outcomes, depression, clinical outcomes, cardiovascular disease, clinical outcomes, exercise outcomes, clinical outcomes, nutrition, clinical outcomes, quality of life, quality, performance improvement, research, quantitative methods, regression

## Abstract

**Introduction:** Weight gain is a risk factor for poor clinical outcomes following kidney transplantation. **Research Question:** This study’s aim was a first testing of 2 models to identify patients early after kidney transplantation who are at risk for weight gain and increase in adipose tissue mass in the first year after kidney transplantation. **Design:** The literature-based models were evaluated on longitudinal data of 88, respectively 79 kidney transplant recipients via ordinary and Firth regression, using gains ≥ 5% in weight and adipose tissue mass respectively as primary and secondary endpoints. **Results:** The models included physical activity, smoking cessation at time of kidney transplantation, self-reported health status, depressive symptomatology, gender, age, education, baseline body mass index and baseline trunk fat as predictors. Area under the curve was 0.797 (95%-CI 0.702 to 0.893) for the weight model and 0.767 (95%-CI 0.656 to 0.878) for the adipose tissue mass model—showing good, respectively fair discriminative ability. For weight gain ≥ 5%, main risk factors were smoking cessation at time of transplantation (OR 16.425, 95%-CI 1.737-155.288) and better self-reported baseline health state (OR 1.068 for each 1-unit increase, 95%-CI 1.012-1.128). For the adipose tissue mass gain ≥ 5%, main risk factor was overweight/obesity (BMI ≥ 25) at baseline (odds ratio 7.659, 95%-CI 1.789-32.789). **Conclusions:** The models have potential to assess patients' risk for weight or adipose tissue mass gain during the year after transplantation, but further testing is needed before implementation in clinical practice.

## Introduction/Background

Weight gain is common after kidney transplantation and a risk factor – independent of Body Mass Index (BMI) range – for poor clinical outcomes including new onset diabetes,^1^ graft loss^2^ and death.^3^ Even in patients with normal pretransplant weight, unmonitored slow weight gain can lead to overweight or obesity.^4^ Therefore, in clinical practice, it is essential to identify patients at risk as early as possible after transplantation.

The underlying assumption of most body weight measurement is that increases in body weight are caused by body fat gain and therefore undesirable. They may also result from increased muscle mass, which is clinically beneficial (eg, for lowering the risk of diabetes); therefore, body weight measurement functions only as a proxy for changes in body composition. To distinguish between fat and other body tissues, fast, non-intrusive methods of calculating body composition have increased in popularity.^5^ For routine clinical or home monitoring, low-cost, reasonably accurate methods, particularly bioelectrical impedance analysis (BIA), are generally more feasible.

Whereas risk factors for body fat gain have been rarely investigated in the first year after kidney transplantation, body weight gain has been explored in numerous prospective and retrospective studies over last 2 decades.^6^ Despite extensive research, a gap in knowledge still exists regarding various factors” impacts on weight gain. While sociodemographic and biomedical factors have been broadly investigated in longitudinal studies after kidney transplantation, few of these have investigated the impact of behavioral,^7,8^ psychological^8,9^ and environmental factors ^10^ on first year posttransplant weight gain; other behavioral factors known to contribute to weight gain in the general population, such as smoking cessation,^11^ simply have not been investigated in kidney transplantation.

Further, it is striking that newer and older studies investigating the impact of certain factors on weight gain after kidney transplantation so often achieve inconclusive results. For example, younger age, ie, age in years at time of inclusion in the study, shows an effect on first-year posttransplant weight gain in some studies,^8,12–14^ but not in others.^10,15,16^ These incongruities might result from methodological differences (eg, analysis applying multivariate models vs comparisons of means); however there may also be moderating factors whose effects are not yet fully understood (eg, interactions between gender and medication) and that may contribute to this variation. In behavioral or psychological variables, differences in operationalization may contribute to the results” heterogeneity. The use of non-standard measurement times (months before transplantation instead at time of transplantation) for predictors of posttransplant weight gain may reduce precision in the prediction model while masking potentially relevant factors.

Although weight gain is known to be a multifactorial phenomenon, few researchers have investigated sociodemographic, biomedical, behavioral, and psychological factors simultaneously in a single study. Based on the existing body of evidence, our aim was to test 2 literature-based models by exploring selected factors of weight gain and adipose tissue gain across sociodemographic, behavioral, psychosocial, and biomedical domains.

## Design/Methods

### Design

The design was prediction model testing. The models were fitted using data from a previously performed prospective randomized control trial (RCT) that tested a repeated behavioral intervention during the first 8 months posttransplant versus a single educational session (usual care).^17^ Variables for this secondary analysis were selected based on the literature and expert opinion. The selection is described in detail elsewhere.^6^

The previously performed RCT was approved by the ethics committee and informed consent was obtained from all individuals included in the study. The study was performed in full compliance with the Helsinki Declaration of 1975, as revised in 2013. The reporting of this study followed the TRIPOD Checklist for Prediction Model Development and Validation.^18^

### Population

The sample was drawn from all patients who got a renal transplantation between May 2012 and February 2018 at the University Hospital Zurich Switzerland. The population was a typical Swiss transplant population. Data about the Swiss transplant population is accessible in publications of the Swiss Transplant Cohort Study (STCS). In the annual report of the STCS 2018, 85.1% of all enrolled kidney-transplanted patients in Switzerland between May 2008 and December 2018 85.1% received their first kidney transplant, mean age was 54.3 (IQR 42.5, 63.1), and 63.7% were male.^19^

### Sampling

For this secondary analysis, we used the original study's sample of 123 participants. Patient files lacking data for either of the endpoints (at either 2-6 weeks or 12 months posttransplant) were excluded from analysis. No imputation was performed for missing data.

The inclusion criteria for the original RCT were age ≥ 18 and having received a kidney transplant in the past 6 weeks. Exclusion criteria were: 1) inability to speak or read German or Italian; 2) previous or combined lung, liver or heart transplantation.

### Data Collection

#### Outcome Variables

The endpoint of this secondary analysis was weight gain at 12 months posttransplant ≥ 5% above baseline in patients with BMI ≥ 18.5 (meets criteria = 1) versus < 5% weight gain (does not meet criteria = 0).

Height and weight were measured at the hospital on a calibrated scale with light clothing (underwear and shirt, without trousers and shoes). The change in body weight was expressed as percentage of change from baseline weight and was calculated as follows: 100 / body-weight-kg_baseline_ * (body-weight-kg_month12_ – body-weight-kg_baseline_). The binary outcome was weight gain <5% from baseline (including weight loss as well) (0) versus weight gain ≥5% from baseline (1). Because weight gain is a clinically desired outcome in underweight patients, only patients with BMI ≥18.5 were included for this outcome.

A second endpoint was adipose tissue mass (ATM) gain at 12 months posttransplant ≥ 5% from baseline (all BMI) (1) versus ATM gain of less than 5% (0). Adipose tissue mass was measured in-hospital via body impedance analysis (Fresenius^TM^), calculations are based upon the following equation: adipose tissue mass (kg) = body weight (kg) - lean tissue mass (kg) - overhydration (liters). Adipose tissue mass change was expressed as percentage of change from baseline ATM: 100/ATMkg_baseline_ * (ATMkg_month12_ - ATMkg_baseline_).

The binary outcome was < 5% ATM gain from baseline (ATMkg) (including ATM loss) (0) versus ≥ 5% ATM gain from baseline (ATMkg) (1). For this outcome, all patients (including underweight ones) were included.

#### Predictor Variables

The same variables were included for changes in both weight and ATM models. Baseline sociodemographics. Age (continuous: variable, difference between inclusion date and birth date) and gender (dichotomous: female = 0, male = 1) were retrieved from the electronic patient charts. Education was assessed by interview (dichotomy: 0 = other than basic education, 1 = basic education). Basic education was defined as ≤9 years of education (mandatory schooling).

**Physical activity**. Cardiovascular-relevant physical activity was assessed as moderate or vigorous activity over the last 7 days via the International Physical Activity Questionnaire (IPAQ). A sum of <150 min was counted as inactivity (1); a sum of ≥150 min was counted as activity (0).

**Baseline depressive symptomatology**. Depressive symptomatology at baseline was assessed with the 7-item (possible score range: 0-21) Depression subscale of the Hospital Anxiety and Depression Scale (HADs).^20^ The HADS is a 14-question self-report instrument that measures anxiety and depression in adults with physical complaints. A high score (3) indicates a high expression of anxiety or depression, whereas a low score (0) indicates no expression. An interval-scaled total score from 0 to a maximum of 21 is calculated for each subscale. For this study, only the depression score was used.

**Baseline health status**. Perceived health was assessed via the European Quality of Life Questionnaire 5 dimensions -Visual Analogue Scale (EQ-5D VAS) on a scale of 0–100 (100 indicating excellent health).

**Smoking cessation**. Smoking cessation between time of transplantation and baseline measurement was also assessed via self-report. Based on the literature search, where weight changes are only expected in patients who change their smoking status, a binary outcome was chosen: patients whose smoking status remained unchanged between time of transplantation and baseline measurement -including non-smokers and ongoing smokers ( = 1), versus those whose smoking status changed (start or quit smoking) between time of transplantation and baseline measurement ( = 0). In our sample, no patients started smoking in the time period; therefore, this group included only patients who quit.

**Baseline body mass index**. Body mass index was calculated and dichotomized to differentiate between normal weight (weight analysis: BMI 18.5-24.99; ATM analysis: BMI ≤24.99) and overweight/obesity (BMI≥25). These categories match those of the World Health Organization (WHO) (0 = normal weight at baseline, 1 = overweight/obese at baseline).^21^

**Baseline adipose tissue mass**. Adipose tissue mass was calculated as described above and used as a continuous variable.

**Treatment group assignment**. In the original study, patients were randomized 1:1 to usual (one educational session, then weight self-monitoring) and intervention care (usual care plus 7-8 counseling sessions focusing on weight management and physical activity during the first 8 months). As the inclusion in the intervention group showed a slight effect, although not significant, group allocation was treated as an influencing variable (0 = intervention group, 1 = control group). The intervention is described in detail elsewhere.^17^

### Data Analysis

Descriptive statistics included means (standard deviations) or medians [interquartile ranges] as appropriate for each distribution pattern. Numbers and percentages were reported for categorical variables. Simple logistic regression was used on all variables and dichotomized outcome variables (primary: weight gain ≥5%, secondary: ATM gain ≥5%, for month 12). Then, multiple logistic regression was applied, including all predictor variables and the binary outcome variables as dependent variables (primary: weight gain ≥5%, secondary: ATM gain ≥5% at month 12).

As standard logistic regression indicated complete or near-complete separation of the predictor variables, Firth (bias-reduced) regression was additionally used.^22^ We used binary logistic regression to calculate the probability for each patient that the dependent variable would be equal to 1. The model's discriminative ability was assessed using the area under the receiver operating characteristic curve (AUC), along with its 95% confidence interval. Only patients with full data sets in the outcome or influencing factors were included in this analysis.

Parts of the analyses were performed using R for windows (R Core Team 2018) and the logistf package for R. The remaining parts of the analysis were performed with SPSS 26 (IBM Corp, Armonk, NY).

## Results

### Derivation of the Model

The model was derived from a cohort of 123 patients. Sample characteristics, influencing and outcome variables distributions are presented in Table 1.

**Table 1.** Sample Characteristics and Baseline Distribution of Influencing and Outcome Variables for the Overall Convenience Sample's Weight and ATM Analysis.

	Convenience sample	Weight Analysis	ATM Analysis
	N = 123	N = 88	N = 79
**Sample characteristics**	**Mean (SD)**	**Mean (SD)**	**Mean (SD)**
Age, year	50.2 (13.1)	52.5 (12.3)	51.9 (11.4)
	**N (%)**	**N (%)**	**N (%)**
Gender, male	76 (61.8)	56 (63.6)	49 (62.0)
Nationality			
Swiss	93 (75.6)	66 (75.0)	56 (70.9)
Swiss and Other	10 (8.1)	8 (9.1)	7 (8.9)
Other	20 (16.3)	14 (15.9)	16 (20.3)
Kidney transplantation	120 (97.6)	87 (98.9)	78 (98.7)
Kidney-pancreas transplantation	3 (2.4)	1 (1.1)	1 (1.3)
First transplantation	98 (79.7)	69 (78.4)	62 (78.5)
Hypertension	54 (43.9)	41 (46.6)	38 (48.1)
Diabetes at time of transplantation	14 (11.4)	8 (9.1)	9 (11.4)
Patients with steroid free regimen	5 (4.1)	3 (3.4)	3 (3.8)
	**Mean (SD)**	**Mean (SD)**	**Mean (SD)**
Baseline Prednisone mg / day	20.1 (8.9)	20.7 (9.4)	21.4 (9.7)
Creatinine at baseline μmol / l	141.8 (50.5)	145 (53.9)	145 (56.6)
eGFR at baseline ml / min	49.9 (15.9)	48.3 (15.5)	48.6 (15.5)
Urea at baseline mmol / l	10.2 (4.2)	10.2 (4.1)	10.0 (4.3)
Haemoglobin at baseline g / l	107.7 (17.1)	107.0 (17.3)	107.0 (17.3)
Aetiology	**N (%)**	**N (%)**	**N (%)**
Diabetes Type 1 or 2	7 (5.7)	3 (3.4)	3 (3.8)
Hypertensive kidney disease	9 (7.3)	8 (9.1)	7 (8.9)
Glomerulonephritis	43 (35.0)	27 (30.6)	26 (32.9)
Polycystic kidney disease	28 (22.8)	23 (26.1)	17 (21.5)
Congenital genetic kidney disease	12 (9.8)	9 (10.2)	7 (8.9)
Genetic (others than polycystic)	7 (5.7)	5 (5.7)	6 (7.6)
Unknown	15 (12.2)	11 (12.5)	11 (13.9)
Others	2 (1.6)	2 (2.3)	2 (2.5)
**Influencing variables at baseline**	**N (%)**	**N (%)**	**N (%)**
Baseline BMI			
Lower than 18.5	9 (7.3)	0 (0.0)	6 (7.6)
18.5 − 24.99	52 (42.3)	35 (39.8)	26 (32.9)
> 25.00	62 (50.4)	53 (60.2)	47 (59.5)
	**Mean (SD)**	**Mean (SD)**	**Mean (SD)**
Baseline ATM (kg)			
Total	25.4 (14.3)	27.1 (13.9)	27.1 (13.4)
Male	24.9 (13.2)	26.4 (12.5)	26.8 (12.8)
Female	26.1 (16.0)	28.4 (16.2)	27.4 (14.5)
Missing, N (%)	8 (6.5)	0 (0.0)	0 (0.0)
	**N (%)**	**N (%)**	**N (%)**
Education			
Basic: ≤9 years of education	11 (8.9)	8 (9.1)	8 (10.1)
> 9 years of education	112 (91.1)	80 (90.9)	71 (89.9)
Smoking *			
Smoking cessation at time of transplant	8 (6.5)	6 (6.8)	4 (5.1)
Others	108 (87.8)	82 (93.2)	75 (94.9)
Missing	7 (5.7)	0 (0.0)	0 (0.0)
Self-reported activity at baseline Inactivity: Less than 150 min of cardiovascular activity per week	65 (52.8)	48 (54.5)	43 (54.4)
Activity: 150 or more minutes of cardiovascular activity per week	58 (47.2)	40 (45.5)	36 (45.6)
	**Mean (SD)**	**Mean (SD)**	**Mean (SD)**
Baseline HADS depression score	2.8 (2.6)	2.8 (2.7)	2.6 (2.6)
Baseline EQ-5D VAS score	71.1 (16.2)	71.2 (16.3)	71.2 (16.5)
	**N (%)**	**N (%)**	**N (%)**
Treatment group assignment			
Intervention group	61 (49.6)	45 (51.1)	38 (48.1)
Control group	62 (50.4)	43 (48.9)	41 (51.9)
**Outcome Variables**			
Weight gain			
<5%	85 (69.1)	65 (73.9)	58 (73.4)
≥5%	38 (30.9)	23 (26.1)	21 (26.6)
ATM gain			
<5%	37 (30.1)	29 (33.0)	32 (40.5)
≥5%	60 (48.8)	44 (50.0)	47 (59.5)
Missing	26 (21.1)	15 (17.0)	0 (0.0)

*smoking status in pre-transplant assessment: 93 non-smokers / 30 smokers: 15 stopped smoking between pre-transplant assessment and baseline measurement (7 time point missing, 8 at time of transplantation) and 15 continued to smoke during the first year posttransplant

HADS, Hospital Anxiety and Depression Scale

EQ-5D VAS, European Quality of Life Questionnaire 5 dimensions -Visual Analogue Scale

From the initial sample of 123 patients, data sets of 88 patients with no missing values were included in the weight analysis (9 were underweight; 26 had at least one detail missing from their data sets) and 79 in the ATM analysis (44 had at least 1 detail missing from their data sets). The ATM analysis included all 73 patients included in the weight analysis plus 6 who were underweight.

### Simple Logistic Regression

The results of the simple logistic regression are presented for gains in overall weight and in ATM (Table 2).

**Table 2.** Risk Factors for Weight Gain and Adipose Tissue Mass Gain at Month 12 via Univariate Binary Logistic Regression.

Risk factor	Weight gain ≥ 5% in patients with BMI ≥ 18.5 at month 12 (N = 88)			ATM gain ≥ 5% at month 12 (N = 79) (all BMI categories included)		
Risk factor	Odds Ratio *Exp(B)*	95% CI for Odds Ratio		Odds Ratio *Exp(B)*	95% CI for Odds Ratio	
Higher age (at baseline), per year	0.983	0.946	1.022	1.001	0.962	1.041
Gender (male)	0.665	0.252	1.757	0.614	0.238	1.579
Lower education (at baseline)	1.800	0.394	8.215	1.151	0.255	5.197
Inactivity, with IPAQ (at baseline)	0.879	0.339	2.279	0.714	0.288	1.770
Smoking cessation at time of transplantation	3.100	0.579	16.597	2.114	0.210	21.280
Overweight or obesity (BMI ≥ 25) (at baseline)	0.237	0.087	0.649	1.937	0.773	4.856
Higher ATM (fat mass) (at baseline), per unit	0.972	0.937	1.009	0.974	0.941	1.009
Higher depressive symptomatology, with HADS (at baseline), per unit	0.906	0.741	1.108	1.117	0.922	1.352
Higher health state, with EQ-5D VAS (at baseline), per unit	1.037	1.001	1.073	1.003	0.976	1.031
One educational session after transplantation (vs monthly behavioral sessions during the first 8 months after transplantation)	1.196	0.462	3.100	0.745	0.302	1.838

ATM, adipose tissue mass

EQ-5D VAS, European Quality of Life Questionnaire 5 dimensions -Visual Analogue Scale

HADS, Hospital Anxiety and Depression Scale

IPAQ, International Physical Activity Questionnaire

### Multiple Logistic Regression: Prediction of 5% Weight Gain (from Baseline Weight) at Month 12

The weight gain model showed good discriminative ability, with an area under the ROC curve of 0.797 (95%-CI 0.702 to 0.893). Nagelkerke R Square was 0.308 and Omnibus Test of Model Coefficient was significant (Chi-Square (df 10) = 20.789, *P* = 0.023).

Risk factors for weight gain include smoking cessation at time of transplantation, which increases the risk by an odds ratio of 16.425 (95%-CI 1.737-155.288), and better EQ-5D VAS health rating, which increases the risk by an odds ratio of 1.068 (95%-CI 1.012-1.128) for each 1-unit increase (total of 100 units). A BMI ≥ 25 at time of transplantation (95%-CI 0.036–.746) was protective against overall weight gain (though not against ATM) (Table 3). The analyses conducted using Firth regression showed comparable results (data not shown).

**Table 3.** Multiple Binary Logistic Regression for Weight Gain of ≥5% (N = 88).

	Estimate	Odds Ratio *Exp(Estimate)*	95%-CI for Odds Ratio		*P*-Value
Intercept	−4.640	0.010			0.066
Higher age (at baseline), per year	−0.021	0.979	0.934	1.026	0.381
Gender (male)	0.125	1.133	0.321	3.997	0.846
Lower education (at baseline)	1.254	3.503	0.572	21.437	0.175
Inactivity, with IPAQ (at baseline)	−0.144	0.865	0.246	3.041	0.822
Smoking cessation at time of transplantation	2.799	16.425	1.737	155.288	0.015
Overweight or obesity (BMI ≥ 25) (at baseline)	−1.808	0.164	0.036	0.746	0.019
Higher ATM (fat mass) (at baseline), per unit	0.004	1.004	0.954	1.056	0.888
Higher depressive symptomatology, with HADS (at baseline), per unit	0.087	1.090	0.810	1.468	0.568
Higher health state, with EQ-5D VAS (at baseline), per unit	0.066	1.068	1.012	1.128	0.017
One educational session after transplantation (vs monthly behavioral sessions during the first 8 months after transplantation)	0.095	1.100	0.354	3.410	0.870

**Outcome variables:** weight gain <5% from baseline (0), weight gain ≥5% from baseline (1); ATM gain < 5% from baseline (ATMkg) (including ATM loss) (0), ATM gain ≥ 5% from baseline (ATMkg) (1); **Influencing Variables:** female (0), male (1); years of education > 9 years (0), ≤9 years of education (basic) (1); activity: ≥150 min moderate or vigorous activity over the last seven days (0), inactivity: <150 min moderate or vigorous activity over the last seven days (1); no change in smoking status between time of transplantation and baseline measurement (including non-smokers and ongoing smokers) (0), smoking cessation between time of transplantation and baseline measurement (1); BMI < 25 at baseline (0), BMI ≥ 25 (1); intervention group (0), control group (1); age (continuous); depressive symptomatology (continuous from 0-21, 21 indicating high depressive symptomatology); health state with (continuous from 0-100); ATM (continuous)

IPAQ, International Physical Activity Questionnaire

ATM, adipose tissue mass

HADS, Hospital Anxiety and Depression Scale

EQ-5D VAS, European Quality of Life Questionnaire 5 dimensions -Visual Analogue Scale

To summarize, based on this regression model, it is possible to identify patients with low probability for weight gain. To illustrate the model's use with real patients, it is useful to consider two actual patient risk constellations – the risk of weight gain for the first being low, the second high. The patient A (Identification-Number 103) was 55 years old, female (0), had more than 9 years of education (0), reported inactivity at baseline (1) and no smoking cessation at time of transplant (0), was overweight/obese at baseline (BMI > 25) (1), an ATM of 26.5%, a very low depressive symptomatology (HADS score: 1/21), and an overall low (40/100) perceived health rating with EQ VAS and was a member of the intervention group (0). Her predicted probability for weight gain ≥ 5% was 0.01, or 1%.

The high-risk patient B (Identification-Number 109) was 63 years old, female (0), had received higher education (0), reported inactivity at baseline (1) and smoking cessation at time of transplantation (1), had a normal baseline BMI (18.5-25) (0) with 15.9% ATM, a HADs score indicating low depressive symptomatology (4/21) and an EQ–VAS perceived health score of 75/100, and was a member of the control group (1). Her predicted probability of first-year weight gain ≥ 5% was 0.90, or 90%.

### Multiple Logistic Regression: Prediction of 5% Adipose Tissue Mass Gain (from Baseline ATM) at Month 12

The ATM gain model's discriminative ability was fair with 0.767 (95%-CI 0.656 to 0.878). Nagelkerke R Square 0.277 and Omnibus Test of Model Coefficient was not significant (Chi-Square (df 10) = 18.164, *P* = .052).

The one identifiable risk factor for ATM gain of ≥5% was overweight/obesity (BMI ≥ 25) at baseline (odds ratio 7.659, 95%-CI 1.789-32.789). Higher ATM at baseline functioned as a protective factor against ATM gain of ≥5%, with the odds ratio decreasing by 0.930 (95%-CI 0.883–.981) for each 1-unit increase in ATM (range 0.6-71.2 units) (Table 4). Furthermore, male gender was a protective factor (odds ratio 0.301, 95%-CI 0.095-0.953).The analyses conducted using Firth regression showed comparable results (data not shown).

**Table 4.** Multiple Binary Logistic Regression for Adipose Tissue Mass Gain of ≥5% (N = 79).

	Estimate	Odds Ratio *Exp(Estimate)*	95%-CI for Odds Ratio		*P*-Value
Intercept	−0.093	0.912			0.968
Higher age (at baseline), per year	−0.011	0.989	0.940	1.040	0.667
Gender (male)	−1.201	0.301	0.095	0.953	0.041
Lower education (at baseline)	0.400	1.491	0.230	9.666	0.675
Inactivity, with IPAQ (at baseline)	−0.078	0.925	0.306	2.789	0.889
Smoking cessation at time of transplantation	1.107	3.026	0.200	45.807	0.424
Overweight or obesity (BMI ≥ 25) (at baseline)	2.036	7.659	1.789	32.789	0.006
Higher ATM (fat mass) (at baseline), per unit	−0.072	0.930	0.883	0.981	0.007
Higher depressive symptomatology, with HADS (at baseline), per unit	0.281	1.325	0.992	1.769	0.057
Higher health state, with EQ-5D VAS (at baseline), per unit	0.028	1.029	0.988	1.072	0.173
One educational session after transplantation (vs monthly behavioral sessions during the first 8 months after transplantation)	−0.360	0.698	0.235	2.073	0.517

IPAQ, International Physical Activity Questionnaire

ATM, adipose tissue mass

HADS, Hospital Anxiety and Depression Scale

EQ-5D VAS, European Quality of Life Questionnaire 5 dimensions -Visual Analogue Scale

The ATM prediction model can identify patients with very low and very high probabilities of ATM gain. For example, patient C (Identification-Number 57) had the following constellation of risk factors: age 39; male (1); higher education (0); inactivity at baseline (1); no smoking cessation at time of transplantation (0); BMI < 25 (0) and 28.1% ATM at baseline; no depressive symptomatology (HADs depression score: 0/21); an EQ VAS score of 50 (of a total of 100); and was in the control group (1). His probability of ATM gain ≥ 5% was 0.06, or 6%.

In contrast, patient D (Identification-Number 87) had a high-risk factor constellation: age 51; male (1); higher education (0); inactivity at baseline (1), no smoking cessation at time of transplantation (0); BMI>25 (1); 18.1% ATM; a rather high HADs depression score of 12/21 (indicating moderate depressive symptomatology), an EQ-VAS score of 50, and was in the control group (1). His predicted probability for ATM gain ≥ 5% was 0.96, or 96%.

## Discussion

Based on an extensive literature review, we developed 2 prediction models for weight gain and body fat gain. These models were tested in a data set of a kidney transplant patient cohort. The models had fair to good discriminative ability, supporting their validation in future clinical studies.

Cessation of smoking at time of transplantation was identified as the main risk factor for weight gain in the early posttransplant period. In our sample, 6.5% (8 of 123) chose their transplantation as the time to quit smoking. The transplantation period was also found to be conducive time to quit smoking in an older study.^23^ Motivation to stop smoking may be extraordinarily high because transplantation indicates a second chance and patients are aware that the kidney may not last forever,^24^ which may increase their sense of obligation to care for themselves and their grafts. Higher perceived health status was also highly influential, but not symptoms of depression. This was congruent with previous studies in kidney transplant recipients.^8,9^

Overweight or obesity (BMI >25) at time of transplantation was protective against weight gain, but a risk factor for increased ATM. Earlier studies” results regarding the impact of baseline BMI on weight gain were controversial: whereas 2 longitudinal studies found that lower BMI increased the risk for weight gain,^15,25^ an older study reported that patients with higher BMIs were at higher risk for weight gain.^12^ Our finding of a lack of overall weight gain but increase in ATM in patients overweight or obese at time of transplantation suggests a greater likelihood of increase in fat mass rather than in lean muscle mass.^8^

Lower education was a risk factor for both overall weight and ATM gain; however, as in Beckmann et al's recently published review of liver transplantation studies, 95%-CI ranges were broad.^26^ Although education might be an important explanatory factor for weight and ATM gain, it is also possible that it is outmoded as a proxy for socioeconomic status, as it reflects persons” early life situations, not necessarily their current ones.

Counter to expectations, inactivity was not a risk factor for gains either in weight or in ATM. This result must be interpreted with caution, as we used self-report measures with no predictive value regarding weight change^8^; one study using pedometers detected an association between daily step counts and weight gain,^7^ whereas another did not.^27^ Also, inactivity 2–6 weeks after renal transplantation may not reflect patients” habitual behavior, but the extraordinary circumstance of recovery from major surgery. Therefore, we suggest further testing of the model with inactivity measured via objective measures for the first year after kidney transplantation.

The discriminative ability of the ATM model was slightly lower than that of the weight model. As the body of evidence on which we based the 2 models referred almost exclusively to overall weight gain, this was not unexpected. The ATM model indicated that female gender and baseline overweight/obesity were predictors for ATM gain. This supported previous findings that patients with high baseline ATM and females have a higher risk for increased adipose tissue.^28^

This study has some limitations. Firstly, our models have not been validated in an external cohort yet. Normally, the AUCs of a validation study are expected to be inferior to those of the derivation study. The reasons are manifold, but in this study, the most eminent reason may be due to optimism in the estimated regression coefficients. However, as the variables selected were literature-based and not based on data, the problem may be less pronounced and reduced the optimism in the coefficients. Secondly, the number of events per category were rather small for some influencing variables, which may impact the models” robustness. Thirdly, our database did not include data on genetic factors or income, relevant factors based on our literature search. They may add further explanatory value for weight gain. And last, our study cohort might not represent transplant patients in other countries, which limits the generalizability of the prediction models.

As we were interested in early risk factors and baseline prednisone dosage was not consistently described as a risk factor in the literature, we did not include the prednisone dosage in the model. If risk factors over the course of the year are of interest, prednisone, especially its cumulative intake over the year, may be considered a risk factor.

Provided that further testing confirms our findings, this study has direct practical applications. The 2 models may help to identify patients at risk for weight gain early after transplantation. Most variables – except body impedance analysis and weight – were assessed via self-report, which involves low effort and cost, making it promising for daily clinical application. Perhaps more importantly, overweight and obese patients are at risk not for weight gain, but for increases in ATM—a risk factor for new onset diabetes.^29^ In particular, visceral adipose tissue content has been indicated as a strong predictor of new-onset diabetes after transplantation.^30,31^ For female patients, the problem was more pronounced. These findings may indicate that overweight and obese patients, especially if female, should be referred to tailored weight management programs (including physical activity and nutrition), whereas further patient characteristics eg preexisting diabetes should be taken into account when delivering the intervention.^32^

Both smoking and weight gain were risk factors for cardiovascular disease; therefore, they must be addressed determinedly.^33^ Even in lung transplantation, the posttransplantation resumption rate was considerable (4%), whereas a short gap between cessation and transplantation has been reported as a risk factor.^34^ Consequently, the patients in our cohort who stopped smoking at time of transplantation may be at high risk for resumption, to which undesired weight gain may add an additional risk factor. Therefore, implementation of smoking cessation programs that address transplant-specific psychological and medical factors, while combining smoking cessation strategies with nutrition counseling, is of the utmost importance.

## Conclusions

Literature-based prediction models showed fair to good discriminative ability to predict weight gain and ATM increase in the first year posttransplant. Further validation in independent cohorts is needed before implementation in clinical practice. A potentially major strength of our model was that it included risk factors of the early posttransplant period, allowing early and easy detection of patients at risk. One strongly suggested action would be to offer early at-risk patients tailored interventions, eg transplant-specific smoking cessation programs that include nutrition counseling.
